# Gamification of POCUS: Are Students Learning?

**DOI:** 10.5811/westjem.2022.11.57730

**Published:** 2023-02-22

**Authors:** Frances M Russell, Daniela Lobo, Audrey Herbert, Joshua Kaine, Jenna Pallansch, Pamela Soriano, JD Adame, Robinson M Ferre

**Affiliations:** Indiana University School of Medicine, Department of Emergency Medicine, Indianapolis, Indiana

## Abstract

**Introduction:**

While gamification of point-of-care ultrasound (POCUS) is well received by learners, little is known about the knowledge gained from material taught during these events. We set out to determine whether a POCUS gamification event improved knowledge of interpretation and clinical integration of POCUS.

**Methods:**

This was a prospective observational study of fourth-year medical students who participated in a 2.5-hour POCUS gamification event consisting of eight objective-oriented stations. Each station had one to three learning objectives associated with the content taught. Students completed a pre-assessment; they then participated in the gamification event in groups of three to five per station and subsequently completed a post-assessment. Differences between pre- and post-session responses were matched and analyzed using Wilcoxon signed-rank test and Fisher’s exact test.

**Results:**

We analyzed data from 265 students with matched pre- and post-event responses; 217 (82%) students reported no to little prior POCUS experience. Most students were going into internal medicine (16%) and pediatrics (11%). Knowledge assessment scores significantly improved from pre- to post-workshop, 68% vs 78% (*P*=0.04). Self-reported comfort with image acquisition, interpretation, and clinical integration all significantly improved from pre- to post-gamification event (*P*<0.001).

**Conclusion:**

In this study we found that gamification of POCUS, with clear learning objectives, led to improved student knowledge of POCUS interpretation, clinical integration, and self-reported comfort with POCUS.

## INTRODUCTION

While the definition of gamification varies, the overarching theme refers to the use of game elements and logistics in traditionally non-game contexts.^1^ This idea has been used in business to motivate employees, as a marketing strategy for sales, and is now becoming more popular in education.^2^,^3^ The goal of gamification in education is to increase learner engagement and improve learning outcomes.^4^ Gamification has been shown to improve learner engagement in medical education and has high learner-satisfaction rates.^5^ A recent review concluded that gamification is “a promising tool to improve learning outcomes by strengthening learning behaviors and attitudes towards learning.”^5^ Examples are widespread within a variety of contexts and specialized training including internal medicine, surgery, and neurology, among many others.^6^–^8^

Building on the gamification movement, a competitive gamification approach for point-of-care ultrasound (POCUS) was first popularized by the SonoGames competition that began at the Society for Academic Emergency Medicine Annual Meeting in 2012.^9^ The basis of this competitive event was to use games and competition-based learning as a tool for resident ultrasound education. Results from this and other gamification events have shown an increase in resident enthusiasm for POCUS learning.^10^ Despite the popularity of this approach, little is known about the ability of a gamification event to improve POCUS knowledge and skill. Based on the principles of competitive gamification, we created a POCUS learning competition for graduating fourth-year medical students to review pertinent POCUS learning objectives included in the longitudinal POCUS curriculum. The purpose of this study was to determine whether this gamification event improved POCUS knowledge, ability to recognize pathology on ultrasound images, and ability to incorporate POCUS findings into a clinical scenario.

## METHODS

### Study Design

This was a prospective observational cohort study assessing fourth-year medical students’ knowledge of POCUS before and after a gamification event. This event was part of their undergraduate medical education (UME) curriculum, and attendance was required. We collected data on 265 students over eight separate events, held from February–April 2022. This study was deemed exempt by the institutional review board with waiver of informed consent.

A UME longitudinal POCUS curriculum was implemented during the participating students’ second year of medical school. In second year, cardiac with inferior vena cava, aorta, renal, bladder volume, and fluid assessment/focused assessment with sonography in trauma (FAST) ultrasound were a required part of the curriculum. During third-year clerkships students learned ultrasound-guided vascular access, cardiac, lung, first trimester obstetric, aorta and FAST ultrasound. In fourth year, students learned rapid ultrasound in shock and hypotension (RUSH) during their required emergency medicine (EM) rotation.

### Study Protocol

Eight gamification events took place on three separate days over three months. The fourth-year medical school class was divided into eight groups, so that roughly an equal number of students were present during each event. This was a requirement due to COVID-19 social distancing precautions. Each event lasted two-and-a-half hours and covered the same content. Students were divided into groups of three to five and rotated through eight stations. These eight stations covered POCUS topics including physics, first trimester obstetrics, soft tissue, deep venous thrombosis, vascular access, hypotension, lung, and gallbladder. Stations lasted 10 minutes and included a combination of hands-on scanning of live models for gallbladder; homemade soft tissue; and deep venous thrombosis phantoms and Blue Phantom ultrasound simulation (CAE Healthcare Inc, Sarasota, FL) for peripheral intravenous phantoms. In addition, an obstetric simulator Vimedix Ob/Gyn manufactured by CAE was used for first trimester obstetrics, and a SonoSim simulator (SonoSim, Santa Monica, CA) was used for hypotension/RUSH. Each station had one to three learning objectives and instructors covered content regarding indications, POCUS image acquisition, interpretation, and clinical integration (see Table 1).

**Population Health Research Capsule**

What do we already know about this issue?

*Gamification of ultrasound improves learner engagement and enthusiasm for point-of-care ultrasound (POCUS)*.

What was the research question?

*Does gamification improve POCUS knowledge for learners?*

What was the major finding of the study?

*A gamification event significantly improved POCUS knowledge (P=0.04) and self-reported POCUS comfort (P<0.001)*.

How does this improve population health?

*Gamification of POCUS was found to be a useful tool to improve medical student learning and medical decision-making*.

Each station was led by a faculty or fellow instructor with POCUS training. Teams performed required tasks at each station to earn points, with a maximum of 10 points awarded at each station. Two teams with the top scores competed against each other in a final competition, which required answering questions correctly for three clinical scenarios with respective pathologic ultrasound images. The cases included the following: a soft tissue abscess in a patient with an area of erythema and tenderness; pulmonary congestion (>2 B-lines in at least two lung zones bilaterally) in a dyspneic patient with end-stage renal disease; and a positive FAST ultrasound in a trauma patient.

Prior to the gamification event students completed a pre-assessment. The pre-assessment questions included which specialty students were applying to for residency training, and students’ prior experience with POCUS. Using a five-point Likert scale we assessed each student’s comfort level in acquiring, interpreting POCUS images, and incorporating POCUS findings into patient care scenarios. There were nine knowledge questions that covered image interpretation (six questions) including normal, pathology or artifact, and patient management after interpreting images (three questions). Eight questions were multiple choice, and one question was true/false. The post-assessment questions were the same as the pre-assessment questions. This was by design to determine true changes in knowledge and decrease potential confounders associated with image quality or understanding of what the question was asking. Students were not given the answers to the questions. Additionally, we evaluated the students’ learning experience during the event. Assessments were made available by QR codes immediately prior and after the event. We collected data directly into REDCap electronic data capture tools hosted at Indiana University (Research Electronic Data Capture).

### Data Analysis

We analyzed differences between pre- and post-event assessment responses using chi square with *P* <0.05 being significant. We performed all statistical analyses using Vassar Stats (http://vassarstats.net, Poughkeepsie, NY) and Microsoft Excel (Microsoft Corp, Redmond, WA).

## RESULTS

A total of 289 fourth-year medical students participated in the gamification event. We analyzed data on 265 (92%) students who completed both a pre- and post-event assessment. We were missing matched data from 24 students. Most students were planning to go into internal medicine (42, 16%); pediatrics (30, 11%); surgery (29, 11%); and EM (29, 11%) for residency training; 217 (82%) students had limited to no prior hands-on POCUS experience (see Table 2).

Comparing pre- to post-event responses, we found students were more confident in doing the following: acquiring POCUS images, mean 2.56–3.57 on a five-point Likert scale (*P*<0.001); interpreting POCUS images, median 2.52–3.51 (*P*<0.001); and integrating POCUS within a clinical context, median 2.66–3.52 (*P*<0.001). Overall, POCUS knowledge scores improved from pre- to post-gamification event (68% correct to 78% correct, *P*=0.04). (See Table 3 for breakdown by modality.) Knowledge of image interpretation improved from 67% to 78% and clinical integration improved from 69% to 78%. Knowledge in lung ultrasound showed the biggest improvement. Knowledge regarding the ability to differentiate a vein from an artery remained high pre- and post-event. Soft-tissue ultrasound image interpretation and clinical integration of these findings worsened after the event.

Overall, 241 (91%) students rated this learning experience as good to excellent, with three (1%) rating it poor. One hundred ninety-one (72%) students felt the content taught during the gamification event was essential to their future practice, and 25 (9%) felt that the content was not essential to their future practice.

## DISCUSSION

Gamification of POCUS is a teaching method used to engage and motivate learners through competition.^9^ This method has been well received by resident learners who have found that POCUS gamification events are an effective educational experience with high satisfaction.^10^,^11^ Prior studies have shown that gamification as a teaching method improves knowledge; however, this has not been well studied in the field of POCUS. In this study, we found that a POCUS gamification event given to fourth-year medical students improved confidence (*P*<0.001) and knowledge with POCUS (*P*=0.04).

We assessed knowledge gained from a POCUS gamification event through direct comparison of pre- and post-knowledge questions. A prior study designed by Liteplo et al to assess the effectiveness of a POCUS gamification event conducted a post-event assessment of residents and POCUS program directors and found that residents reported their ultrasound knowledge and clinical use of POCUS increased.^10^ The Liteplo study showed how gamification events can increase enthusiasm and potentially improve use of POCUS; however, this prior data was limited by a small sample size and survey methodology used to assess both knowledge and usage.

Lobo et al^11^ evaluated effectiveness from a two-day POCUS gamification event to EM interns through use of a pre- and post-knowledge assessment. They found improved knowledge with a pre- to post-test score difference of 1.19 (*P*<0.05). Lai et al^12^ randomized 31 doctors with two to four years of clinical experience to a gamified arm vs conventional learning. In their study, they found both methods of teaching significantly improved knowledge and skill, with the participants in the gamification arm stating that this method of teaching was useful in motivating them to learn the RUSH examination. The data from these studies was similar to our study results, finding improved POCUS knowledge after a gamification event. However, they were limited by a small sample size, and it is unknown whether the differences in pre- to post-event knowledge was gained in basic technical skill, image interpretation, or clinical integration.

Although we found improvement in self-rated confidence and knowledge with POCUS, not all areas improved. Interestingly, soft-tissue ultrasound knowledge worsened when comparing knowledge before to after the event. The knowledge assessment included a clinical vignette with an image of an abscess with associated cobblestoning: pre- to post-event 63% vs 54% chose the correct response—an abscess, while 22% vs 45% chose cellulitis. While cellulitis was present in the image, it was important for students to recognize an abscess to guide appropriate management of the patient. This led most students to choose antibiotics alone as a treatment option instead of incision and drainage as the correct response for the subsequent question. Soft tissue ultrasound is one of the easier imaging modalities to perform and interpret.^13^ It is possible that students focused on the cobblestoning, which was present in the image, and disregarded the rest of the image, which also included a fluid collection. It is less likely a result of instruction, as abscess was covered in depth during the soft tissue station and the hands-on portion of the station involved students performing an ultrasound-guided abscess drainage. Additionally, abscess was discussed and shown to students during the deep venous thrombosis station.

Knowledge in lung ultrasound showed the biggest improvement: 60% correct to 90% correct for interpretation; and 56% to 79% correct for clinical integration. This is most likely a reflection of the ease of learning, performing, and interpreting lung ultrasound.^14^,^15^ These results suggest that lung ultrasound is a suitable POCUS modality for teaching during a gamification event whereas other modalities, such as soft tissue ultrasound, are more complex and may require more time to learn than a gamification event would allow.

A POCUS gamification event requires a significant amount of time and resources to plan and implement, typically taking months of planning^9^ and multiple scheduled meetings to discuss content, flow of event, instructor education, and development of materials and phantoms. Despite this, we believe it is a worthwhile teaching modality as it was well received by students and residents, as described in this study and others, 10,11 and importantly increased POCUS knowledge.

## LIMITATIONS

There are several study limitations to consider, which will limit its generalizability. We conducted the study at a single medical school with the participants solely comprised of fourth-year medical students. Stations were created and facilitated by faculty and fellows with advanced training in clinical ultrasound. The ability to reproduce these findings will depend on the development of a curriculum with simple, well-defined, and measurable objectives that can be conducted by an array of instructors regardless of their ultrasound training background. Additionally, we did not assess participant psychomotor skills. Although hands-on scanning was involved with every station, some stations used standardized patients, while most stations used phantoms. This, in addition to time restrictions for the event, limited our ability to assess psychomotor skills for each student independently.

We did not assess for retention of knowledge over time. Lastly, following Kirkpatrick’s model of training, this event largely focused on *learning* and *reaction* by determining student satisfaction and knowledge.^16^ While the data from our study adds to the literature by demonstrating how gamification of POCUS can lead to improved knowledge, future studies should focus on psychomotor skills acquired during a gamification event, knowledge retention, and how the knowledge gained from this event impacts future behavior.

## CONCLUSION

We found that gamification of POCUS, with clear learning objectives, led to improved student knowledge and to an increase in self-reported comfort in acquiring and interpreting images as well as incorporating POCUS into clinical practice. This is the first study to our knowledge involving a large cohort of undergraduate medical students in a POCUS gamification event. Over 88% of students reported this experience as good or excellent, and more than 70% of students rated the skills taught during this course as essential to very essential to their future practice. Gamification of POCUS proved to be a useful tool in improving student learning and their medical decision-making based on complementing information acquired clinically and through ultrasound image interpretation.

## Figures and Tables

**Table 1:** Station learning objectives.

Station	Objectives
OB	- Students will be able to identify an IUP on ultrasound
Physics	- Students will be able to identify posterior acoustic enhancement
Soft tissue	- Students will be able to recognize an abscess on ultrasound - Students will be able to determine the correct treatment for a patient with a skin and soft tissue infection based on ultrasound findings
PIV	- Students will be able to differentiate an artery from a vein
Hypotension/RUSH	- Students will be able to identify free intraperitoneal fluid in the RUQ - Students will be able to determine the correct management for a hypotensive patient with a positive FAST
Lung	- Students will be able to identify B-lines on lung ultrasound - Students will be able to determine the correct treatment for a short of breath patient with B-lines
Gallbladder	- Students will be able to identify normal gallbladder anatomy with ultrasound - Students will be able to recognize ultrasound findings of acute cholecystitis
DVT	- Students will be able to identify or name ultrasound characteristics of DVT - Students will be able to identify risk factors for DVT - Students will be able to determine the correct management for DVT

*DVT*, deep venous thrombosis; *FAST*, focused assessment with sonography in trauma; *IUP*, intrauterine pregnancy; *OB*, obstetrics; *PIV*, peripheral intravenous; *RUQ*, right upper quadrant; *RUSH*, rapid ultrasound in shock and hypotension.

**Table 2:** Student demographics.

Future specialty	n (%)
Internal medicine	42 (16)
Pediatrics	30 (11)
Surgery	29 (11)
Emergency medicine	29 (11)
Other	135 (51%)
Level of prior ultrasound experience	
None	9 (3.4)
Some/used a few times	208 (78)
Moderate/use a couple times per month	46 (17)
Large amount/use weekly	1 (0.6)

**Table 3:** Knowledge scores pre- and post-gamification event, N=265.

	Pre	Post	P-value
Physics Able to identify posterior acoustic enhancement	187 (71%)	229 (86%)	<0.001
OB Able to identify a yolk sac within a gestational sac	115 (43%)	176 (63%)	<0.001
OB Able to identify an intrauterine pregnancy	213 (80%)	229 (86%)	0.04
Soft tissue Able to diagnose an abscess	194 (73%)	154 (58%)	0.11
Soft tissue Treat abscess with an incision and drainage procedure	167 (63%)	142 (54%)	<0.001
Vascular access Differentiate a vein from an artery	238 (90%)	249 (94%)	0.22
Hypotension Able to determine correct treatment for hypotensive patient with a +FAST	201 (76%)	242 (91%)	<0.001
Lung Identify B-lines	160 (60%)	239 (90%)	<0.001
Lung Determine the correct treatment for a short-of-breath patient with B-lines	148 (56%)	209 (79%)	0.01

*FAST*, focused assessment with sonography in trauma; *OB*, obstetrics.
